# APOE4 lead to neurovascular dysfunction & inflammation is an early change in Alzheimer’s disease?

**DOI:** 10.1002/alz.095248

**Published:** 2025-01-09

**Authors:** Mun Seong CHOI

**Affiliations:** ^1^ cheonan chungmu hospital, cheonan si Korea, Republic of (South)

## Abstract

**Background:**

Vascular contributions to dementia & Alzheimer’s disease are increasing recognized. Recent studies have suggested that blood‐brain barrier breakdown is an early biomarker of human cognitive dysfunction, including the early clinical stages of AD. Apolipoprotein E4(APOE4), the major AD susceptibility gene, leads to accelerated blood‐brain barrier breakdown & degeneration of brain capillary pericyte that maintain blood‐brain barrier integrity.

APOE4 is associated with a loss of anti‐inflmmatory function and fails to surpress the basal activation of macrophages and microglia.

**Method:**

An screening yielded publcations of which relevant articles were selected after an evaluation of their titles & abstracts. And then full text of these articles was obtained & compared about above respective subjects thoroughly.

**Result:**

Impotantly, postmortem analysis indicated that the BBB breakdown is more pronounced in individuals with AD who carry the APOE4 allele. Astrocyte‐derived human apoE4 leads to an age‐dependent progressive BBB breakdown by activating a proinflammatory CypA‐nuclear factor(NF)‐ κB‐matrix‐metalloproteinase‐9 pathway(MMP‐9) in brain capillary pericytes. The activation of MMP‐9 in APOE4 mice leads to enzymatic degradation of the BBB tight junction & basement membrane proteins resulting in BBB breakdown followed by neuronal uptake of multiple blood‐derived neurotoxic proteins(e.g., thrombin, fibrin), perivascular deposition of erythrocyte‐derived hemosiderin & microvascular & CBF reductions. This leaky blood‐born proteins induce increasing neuroinflmmation & oxidative stress. APOE4 promotes upregulation of proinflammatory cytokines, nitric oxide(NO) release and macrophage cell death in vitro. Astrocyte secreated apoE3 & apoE2, but not apoE4, suppress the CypA‐NF‐ κB‐MMP‐9 pathway in pericytes via the low density lipoprotein receptor related protein1(LRP1). ApoE4 expression is assocated with a significant increase in amyloid plaques in brain at earlier ages compared with apoE3 or apoE2. ApoE4 impairs A β clearance from & across the BBB in animal models & patients at risk for developing AD.

**Conclusion:**

Future studies should explore whether similar early neuroimaging & biochemical markers of BBB disruption and inflammation are present in humans carrying the ApoE4 allele before cognitive decline and/or A β accumulation occur.

